# Ultrathin MWCNT/Ti_3_C_2_T_x_ Hybrid Films for Electromagnetic Interference Shielding

**DOI:** 10.3390/nano15010006

**Published:** 2024-12-25

**Authors:** Chuanxin Weng, Junzhe He, Jiangxiao Tian, Wei Wu, Jinjin Li, Jiulin Zhang, Haitao Yu, Xuechuan Zhang, Mingming Lu

**Affiliations:** National Key Laboratory of Scattering and Radiation, Beijing 100854, China

**Keywords:** carbon nanotube, Ti_3_C_2_T_x_, electromagnetic interference shielding

## Abstract

The disordered assembly and low conductivity of carbon nanotubes are the main problems that limit the application of electromagnetic interference (EMI) shielding. In this work, an ordered lamellar assembly structure of multiwalled carbon nanotube/Ti_3_C_2_T_x_ (MWCNT/Ti_3_C_2_T_x_) hybrid films was achieved by vacuum-assisted filtration through the hybridization of Ti_3_C_2_T_x_ nanosheets and carbon nanotubes, where carbon nanotubes were tightly sticking on the surface of Ti_3_C_2_T_x_ nanosheets via physical adsorption and hydrogen bonding. Compared with the pure carbon nanotubes films, the hybrid MWCNT/Ti_3_C_2_T_x_ films achieved a significant improvement in conductivity of 452.5 S/cm and EMI shielding effectiveness (SE) of 44.3 dB under 50 wt% Ti_3_C_2_T_x_ with a low thickness (8.6 μm) and orderly lamellar stacking structure, which finally resulted in high specific SE (SSE/t, SE divided by the density and thickness) of 55,603.1 dB∙cm^2^∙g^−1^.

## 1. Introduction

With the rapid development of electronics, electronic equipment has increasingly become an indispensable part of daily life. However, unnecessary electromagnetic radiation may be generated by the employment of electronic equipment, and such electromagnetic interference may affect the normal operation of precise instruments and even cause detrimental effects on human health [1,2,3,4,5,6]. Metal materials are the classical EMI shielding materials [7,8,9], such as copper, aluminum, nickel, iron, and other metal materials, which appear to have outstanding EMI shielding performance by preventing the propagation of electromagnetic waves based on excellent conductive or magnetic properties. Nevertheless, the inherent attributes of metal materials, such as high density, poor flexibility, and corrosion resistance, seriously limit the application of metal-based shielding materials. 

In recent years, carbon nanomaterials [10,11,12] have become a new type of EMI shielding material due to their excellent electrical properties and environmental tolerance. Among them, carbon nanotubes have attracted much attention due to their easy mass preparation and their relatively high intrinsic conductivity [13,14,15]. However, it is difficult to acquire well-dispersed carbon nanotubes because they are chemically inert [16]. As a result, complex surface modification has been employed to avoid severe agglomeration, which may increase the contact resistance and result in low conductivity [10,11,16,17]. Generally, the EMI shielding effectiveness is directly determined by the electrical conductivity of materials, where the electromagnetic waves are reflected due to impedance mismatching and absorbed by conduction loss [18]. Therefore, to minimize the electron transmission resistance of carbon nanotubes, reasonable structural design is believed to be one effective strategy for preparing high-performance EMI shielding composites. Particularly, a nacre-like lamellar structure with highly oriented one- or two-dimensional (1D/2D) conductive nanomaterials is supposed to be one of the most promising candidates [18,19,20]. For the 1D/2D composite films with lamellar structure, on the one hand, a conductive pathway would be established with the oriented conductive fillers; on the other hand, the transmission path of waves would be extended significantly by multireflection, which results in enhancing conductivity and electromagnetic wave attenuation with an ultrathin thickness.

Ti_3_C_2_T_x_ (T_x_: surface-terminating functionality (e.g., F, O, and OH)) is a new two-dimensional material like graphene [21,22] possessing excellent electrical properties [23] (conductivity up to 4600 S/cm) and rich surface functional groups (such as some oxygen-containing and fluorine-containing functional groups), which is popular in compounding with other nanomaterials and polymer matrix. The high electrical conductivity of Ti_3_C_2_T_x_ determines its excellent performance in EMI shielding [24]. For instance, Ti_3_C_2_T_x_ films can achieve high EMI SE at relatively low thickness (45 μm, 92 dB@8.2 GHz) [22]. In addition, many EMI shielding composites of Ti_3_C_2_T_x_ and other nano-conductive materials have been manufactured. For example, Liu et al. [25] compounded Ti_3_C_2_T_x_ with conductive polymer poly(3,4-ethylenedioxythiophene)-poly(styrenesulfonate) (PEDOT:PSS) to optimize the mechanical and EMI shielding properties of the composite film by adjusting the mixing ratio of Ti_3_C_2_T_x_ and PEDOT:PSS. Liu et al. [19] sprayed Ti_3_C_2_T_x_ and silver nanowire suspension in turns on a fabric surface, and the EMI shielding performance of the composites was able to reach 46 dB (@8.2 GHz) owing to the synergistic effect of Ti_3_C_2_T_x_ and the silver nanowire conductive network. 

Herein, the Ti_3_C_2_T_x_ nanosheets were employed to assist MWCNT in forming a lamellar assembly structure and further improving the conductivity by a vacuum-assisted filtration method. Furthermore, the obtained MWCNT/Ti_3_C_2_T_x_ hybrid film with a lamellar structure shows a high electrical conductivity of 452.5 S/cm and an enhanced EMI SE of 44.3 dB. The lamellar structure of the hybrid film plays a crucial role in dissipating electromagnetic waves, especially when electromagnetic waves propagate in the lamellar structure, where the synergistic interactions of the MWCNT and Ti_3_C_2_T_x_ would generate high conduction loss and dipole polarization loss to the electromagnetic waves, and multiple reflections of incident electromagnetic waves within the lamellar structure. In contrast to the disordered structure of pure MWCNT films, the lamellar structure of MWCNT/Ti_3_C_2_T_x_ hybrid films possesses more favorable advantages in the improvement of EMI shielding performance. This work thus provides a simple method to realize the ordered assembly of carbon nanotubes with high EMI shielding performance.

## 2. Experimental Section

### 2.1. Materials

The multiwall carbon nanotubes (TNM2, diameter: 8–15 nm, length: 50 μm) were purchased from Chengdu Organic Chemistry Co., Ltd., Chinese Academy of Sciences (Chengdu, China). The MWCNT aqueous suspension with 1 wt% content was obtained by ultrasonic treatment at 400 W for 30 min and magnetic stirring for hours with the aid of non-covalent surfactant (TNWDIS, aromatic modified polyethylene glycol ether, supplied by Chengdu Organic Chemicals Co., Ltd., CAS). Hydrochloric acid (HCl) was provided by Beijing Chemical Works (Beijing, China). Lithium fluoride (LiF) was obtained from Shanghai Aladdin Biochemical Technology Co., Ltd. (Shanghai, China). Ti_3_AlC_2_ powder (400 mesh) was provided by Jilin 11 Technology Co., Ltd. (Changchun, China).

### 2.2. Preparation of Ti_3_C_2_T_x_

Ti_3_C_2_T_x_ was synthesized by a typically selective etching method [26], where 3.2 g LiF was firstly dissolved in 40 mL HCl (9 M) in a Teflon container and further magnetically stirred until completely dissolved. Secondly, 2 g Ti_3_AlC_2_ powder was gradually poured into a container, and the mixture was continuously stirred for 24 h at 35 °C. Afterward, a repeated wash with H_2_O under centrifugation and shaking was employed to prepare the Ti_3_C_2_T_x_ suspension until the pH of the dispersion was near neutral. The concentration of the obtained Ti_3_C_2_T_x_ suspension was about 10 mg/mL.

### 2.3. Preparation of MWCNT/Ti_3_C_2_T_x_ Hybrid Films

The MWCNT suspension and the Ti_3_C_2_T_x_ suspension were diluted to 1 mg/mL with deionized water, and then Ti_3_C_2_T_x_ suspension was added to the MWCNT suspension to prepare a mixed dispersion with different Ti_3_C_2_T_x_ contents (10 wt%, 20 wt%, 30 wt%, 40 wt%, and 50 wt%) by a planetary vacuum mixer (THINKY ARV-310, Osaka, Japan). Subsequently, the MWCNT/Ti_3_C_2_T_x_ hybrid films were fabricated through a simple vacuum-assisted filtration method.

### 2.4. Characterization

The morphologies of Ti_3_AlC_2_ powder, Ti_3_C_2_T_x_ nanosheets, and cross-section of the MWCNT/Ti_3_C_2_T_x_ hybrid films were examined by scanning electron microscope (SEM, Hitachi S4800, Tokyo, Japan). X-ray diffraction (XRD) patterns of Ti_3_AlC_2_ and Ti_3_C_2_T_x_ were measured by a Rigaku SmartLab (Tokyo, Japan) 9 kW XRD (Cu Kα radiation, 40.0 Kv and 200.0 mA) with a scan speed of 10.0 degrees per minute. A high-performance multimeter (KEITHLEY, DMM 7510, Cleveland, OH, USA) was employed to measure the resistance of MWCNT film and MWCNT/Ti_3_C_2_T_x_ hybrid films. 

### 2.5. EMI Shielding Effectiveness 

A waveguide method was employed in this work to measure EMI SE in the frequency range of 8.2–12.4 GHz (X-band) by using a vector network analyzer (Agilent E8363B PNA-L, Santa Clara, CA, USA), where more than four samples were tested for MWCNT/Ti_3_C_2_T_x_ hybrid films. The MWCNT/Ti_3_C_2_T_x_ hybrid films were cut into 22.86 × 10.16 mm^2^ and placed between two Teflon blocks. According to the possible mechanism of the hybrid film, the *R*, *T*, and *A* represented the reflection, transmission, and absorption coefficients, respectively, which were calculated by the *S* parameters. Furthermore, the EMI *SE* was obtained by the following formulas:

(1) $$R={\left|S_{11}\right|}^{2}\;\;T={\left|S_{21}\right|}^{2}\;\;A+T+R=1$$

(2) $$SE_{T}(dB)=-10lgT$$

(3) $$SE_{R}(dB)=-10lg(1-R)$$

(4) $$SE_{A}(dB)=SE_{T}-SE_{R}$$

where the *SE_T_*, *SE_R_*, and *SE_A_* are the total, reflective, and absorptive EMI *SE*, respectively, and the |*S_ij_*|^2^ is the power transferred from port *i* to port *j*.

## 3. Results and Discussion

**Figure 1:**
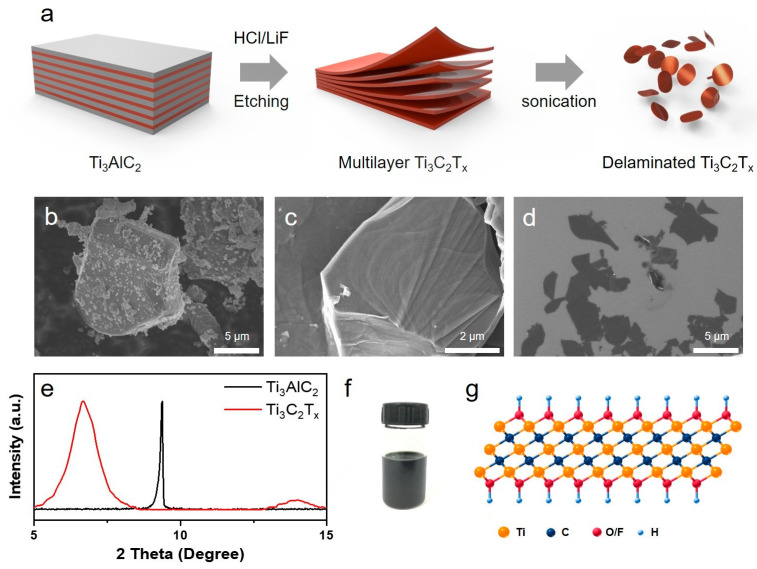
The preparation and characterization of Ti_3_C_2_T_x_ nanosheets. (**a**) Schematic diagram of selective etching for preparing Ti_3_C_2_T_x_ nanosheets. The microstructure of (**b**) Ti_3_AlC_2_, (**c**) multilayer Ti_3_C_2_T_x,_ and (**d**) few-layer Ti_3_C_2_T_x_, respectively. (**e**) XRD patterns for Ti_3_AlC_2_ and Ti_3_C_2_T_x_. (**f**) The Ti_3_C_2_T_x_ aqueous suspension (10 mg/mL). (**g**) Illustration of the atom structure of Ti_3_C_2_T_x_.

As shown in Figure 1a, the schematic diagram presents a classical preparation process of Ti_3_C_2_T_x_ nanosheets. The Al layer of Ti_3_AlC_2_ was etched away by a mixture of lithium fluoride and hydrochloric acid, and then the obtained multilayer Ti_3_C_2_T_x_ was dealt with using an ultrasonic procedure to obtain a few layers of Ti_3_C_2_T_x_ nanosheets. In addition, the corresponding microstructure of the Ti_3_AlC_2_ powder, multilayer Ti_3_C_2_T_x_, and Ti_3_C_2_T_x_ nanosheets are presented in Figure 1b–d, where the Ti_3_AlC_2_ powder showed a blocky structure with a micrometer’s thickness (Figure 1b), and the multilayer Ti_3_C_2_T_x_ presented a loose page-like structure after etching of the Al layer (Figure 1c). After the ultrasonic procedure, the Ti_3_C_2_T_x_ nanosheets were peeled off from the multilayer Ti_3_C_2_T_x_, as shown in Figure 1d, possessing a lateral size distribution with 0.5–6.2 μm. Furthermore, the XRD characterization (Figure 1e) indicates that the 002 crystal surface characteristic peak of Ti_3_AlC_2_ powder moves from 9.4° to 6.7°, which indicates an increase in interlayer spacing when a compacted bulked structure (Ti_3_AlC_2_ powder) turned into loosened nanosheets, which confirmed the successful preparation of Ti_3_C_2_T_x_ nanosheets. As shown in Figure 1f, the obtained Ti_3_C_2_T_x_ exhibited excellent dispersity in aqueous suspension, which is due to a large number of oxygen and fluorine functional groups on the surface of Ti_3_C_2_T_x_ (Figure 1g), which are conducive to further hybridize with other nanomaterials to manufacture multifunctional materials.

**Figure 2:**
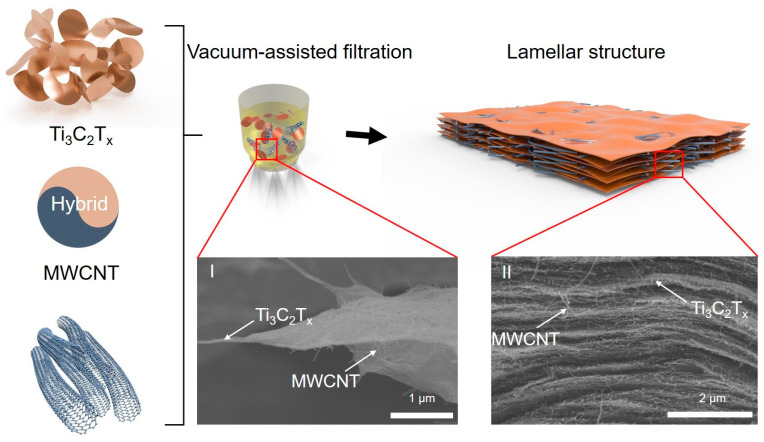
Illustration of the preparation process of the MWCNT/Ti_3_C_2_T_x_ hybrid film through filtration. (I: the microstructure of Ti_3_C_2_T_x_ nanosheets and MWCNT. II: lamellar structure of the MWCNT/Ti_3_C_2_T_x_ hybrid film).

Figure 2 shows the assembly process and microstructure of the MWCNT/Ti_3_C_2_T_x_ hybrid film. The hydrophilic Ti_3_C_2_T_x_ nanosheets with negative surface charge are easily dispersed in an aqueous suspension [27,28]. Meanwhile, the MWCNTs are also well dispersed in an aqueous suspension with the assistance of a non-covalent surfactant. As shown in inset I of Figure 2, the MWCNTs were compactly adsorbed on the Ti_3_C_2_T_x_ nanosheets to form a hybrid structure in the mixed suspension, which may be due to the hydrogen bonding between the Ti_3_C_2_T_x_ nanosheets and MWCNTs. After a vacuum-assisted filtration process, the hybrid Ti_3_C_2_T_x_ nanosheets and MWCNTs’ structure were assembled with a lamellar structure, as shown in inset II of Figure 2, where the Ti_3_C_2_T_x_ nanosheets and MWCNTs can be observed, and the cross-sectional morphology of the MWCNT/Ti_3_C_2_T_x_ hybrid film showed a well-aligned and compacted lamellar structure.

**Figure 3:**
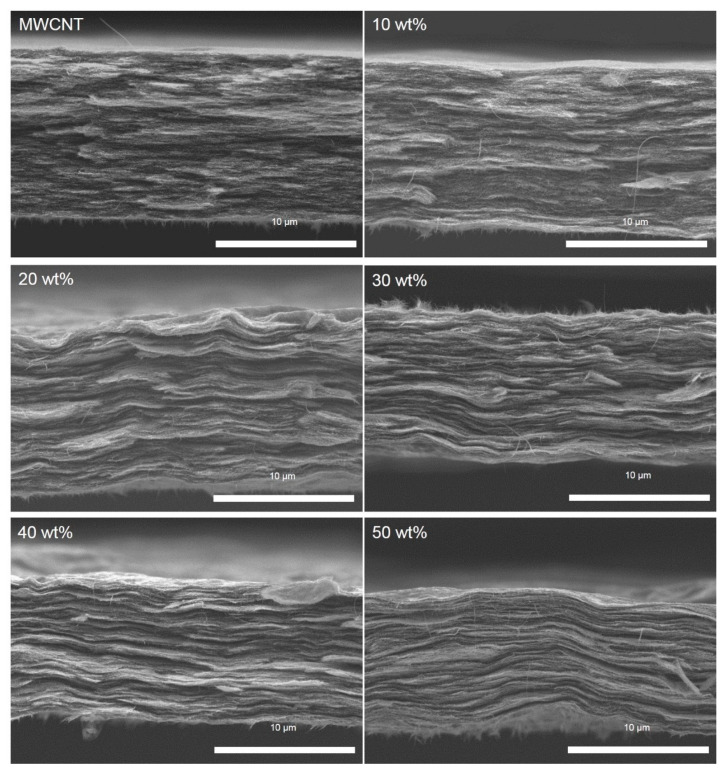
The cross-section of MWCNT and MWCNT/Ti_3_C_2_T_x_ hybrid films under different Ti_3_C_2_T_x_ loading.

A series of MWCNT/Ti_3_C_2_T_x_ hybrid films with different Ti_3_C_2_T_x_ contents were prepared by the vacuum-assisted assembly method as mentioned above. As shown in Figure 3, the cross-sectional morphology of pure carbon nanotube film is mainly composed of the interwinding and overlapping of carbon nanotubes, which exhibits a rough cross-section with pulling out and fracturing of carbon nanotubes. Compared with the random assembly structure of pure carbon nanotube films, the introduction of Ti_3_C_2_T_x_ nanosheets induces an ordered assembly of MWCNT/Ti_3_C_2_T_x_ hybrid films, as shown in Figure 3. When Ti_3_C_2_T_x_ content is 10 wt%, there is no significant difference between MWCNT/Ti_3_C_2_T_x_ hybrid film and pure MWCNT film in the microstructure. With the Ti_3_C_2_T_x_ content increased to 20 wt%, the internal structure of the MWCNT/Ti_3_C_2_T_x_ hybrid film shows a lamellar structure. Furthermore, the microstructure of MWCNT/Ti_3_C_2_T_x_ hybrid film tends to be assembled in an orderly fashion into a lamellar structure with further introducing Ti_3_C_2_T_x_ nanosheets. 

**Figure 4:**
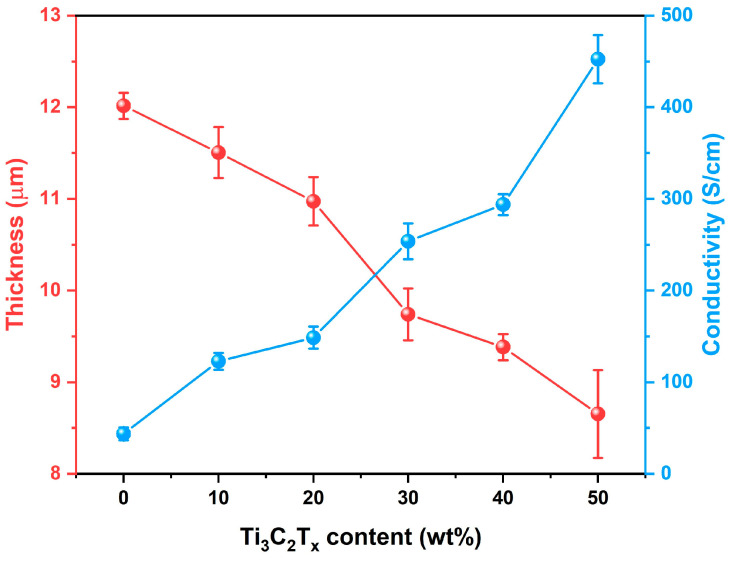
The thickness and conductivity of MWCNT and MWCNT/Ti_3_C_2_T_x_ hybrid films with different Ti_3_C_2_T_x_ contents.

In addition, Figure 4 shows that the thickness of the hybrid films decreases from 12.0 μm (pure carbon nanotube film) to 8.6 μm (50 wt% Ti_3_C_2_T_x_) with the increase in Ti_3_C_2_T_x_ content, which is mainly due to a large number of carbon nanotubes being attached to the surface of Ti_3_C_2_T_x_ resulting in a highly orderly lamellar structure that reduces the space of MWCNTs. Correspondingly, the conductivity of the hybrid film significantly increased from the original 43.6 S/cm (pure carbon nanotube film) to 452.5 S/cm (50 wt% Ti_3_C_2_T_x_), as shown in Figure 4, which is mainly due to the hybrid structure of Ti_3_C_2_T_x_ nanosheets and MWCNTs, where the Ti_3_C_2_T_x_ nanosheets possessed higher electrical conductivity than the carbon nanotubes resulting in more conducive paths constructed in the internal hybrid films.

**Figure 5:**
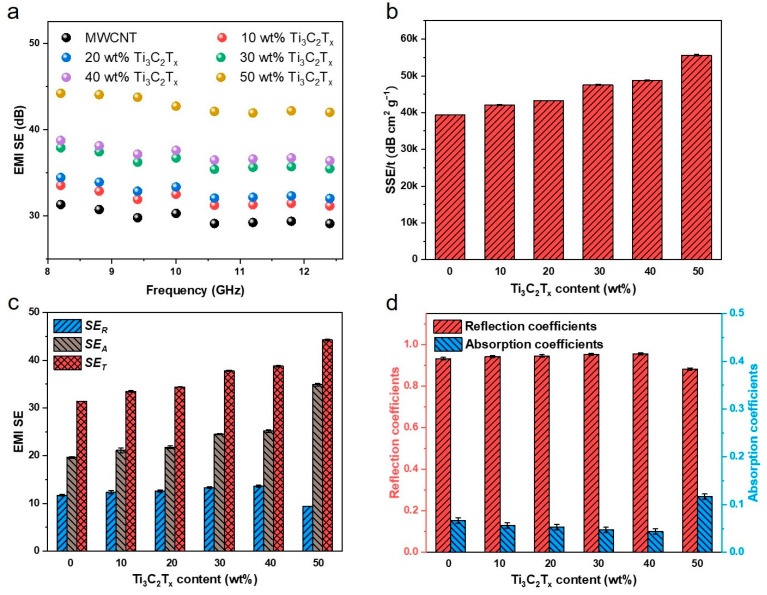
The EMI performance of MWCNT/Ti_3_C_2_T_x_ hybrid films. (**a**) The EMI SE of MWCNT/Ti_3_C_2_T_x_ hybrid films in X-band. (**b**) The specific SE of MWCNT/Ti_3_C_2_T_x_ hybrid films. (**c**) The EMI SE_T_, SE_A_, and SE_R_ of MWCNT/Ti_3_C_2_T_x_ hybrid films. (**d**) The power coefficients R and A value of MWCNT/Ti_3_C_2_T_x_ hybrid films.

The excellent electrical properties of MWCNT/Ti_3_C_2_T_x_ hybrid film give it outstanding performance in EMI shielding performance. As shown in Figure 5a, the EMI SE of pure carbon nanotube film is about 31.3 dB (@8.2 GHz). With the increase in Ti_3_C_2_T_x_ content, the EMI SE of the film is significantly improved. When the Ti_3_C_2_T_x_ content is 50 wt%, the EMI SE reaches 44.3 dB.

As reported in previous work [22,29,30], there is a close relationship between the EMI SE and the thickness and electrical conductivity of films, which are positively correlated factors for its EMI shielding performance. However, the thickness and conductivity of MWCNT/Ti_3_C_2_T_x_ hybrid film show an opposite trend with the increase of Ti_3_C_2_T_x_ content (Figure 4), which is specifically manifested in the EMI shielding performance. As shown in Figure 5a, when the Ti_3_C_2_T_x_ content is low, there is no significant improvement in the EMI shielding performance of MWCNT/Ti_3_C_2_T_x_ hybrid film, but with the further increase of Ti_3_C_2_T_x_ content to 50 wt%, the EMI SE of the hybrid film presents a significant increase. Specifically, on the one hand, with the increase in Ti_3_C_2_T_x_ content, the conductivity of the hybrid films presents rapid growth of a couple of orders of magnitude, but the thickness is without obvious change. On the other hand, the introduction of Ti_3_C_2_T_x_ would urge the hybridization of Ti_3_C_2_T_x_ and MWCNT to assemble with a lamellar structure, indicating that more electromagnetic waves would be dissipated within the stratified structure. In addition, the specific SE (SSE/t, SE divided by the density and thickness) is calculated to evaluate the EMI effectiveness of films, where a high SSE/t value of 55,603.1 dB∙cm^2^∙g^−1^ was achieved under 50 wt% Ti_3_C_2_T_x_ (Figure 5b).

The total EMI shielding effectiveness (SE_T_) can be divided into the absorption of electromagnetic waves (SE_A_) and the reflection from samples (SE_R_). As shown in Figure 5c, the average SE_T_, SE_A_, and SE_R_ (at 8.2 GHz) of MWCNT/Ti_3_C_2_T_x_ hybrid film with different Ti_3_C_2_T_x_ loading contents are listed. As expected, the SE_T_ and SE_A_ increase monotonically with increasing Ti_3_C_2_T_x_ contents. We attribute the effective EMI shielding of MWCNT/Ti_3_C_2_T_x_ hybrid films to the enhanced EMI absorption with the lamellar architecture and the excellent electrical conductivity of the conductive fillers. Furthermore, to evaluate the dissipation energy of electromagnetic waves during the propagation process, the power coefficients of reflectivity (R) and absorptivity (A) are acquired based on the S parameters. As shown in Figure 5d, the reflection coefficients of the MWCNT/Ti_3_C_2_T_x_ hybrid films are about 0.9, indicating that 90% of the electromagnetic wave energy is reflected to the free space, which is due to the impedance mismatch between free space and hybrid films that resulting in the reflection of most electromagnetic waves. Interestingly, the MWCNT/Ti_3_C_2_T_x_ hybrid films (50 wt% Ti_3_C_2_T_x_) exhibited a little increase in their absorption coefficient, which implies the considerable contribution of the lamellar structure to the EMI shielding performance.

**Figure 6:**
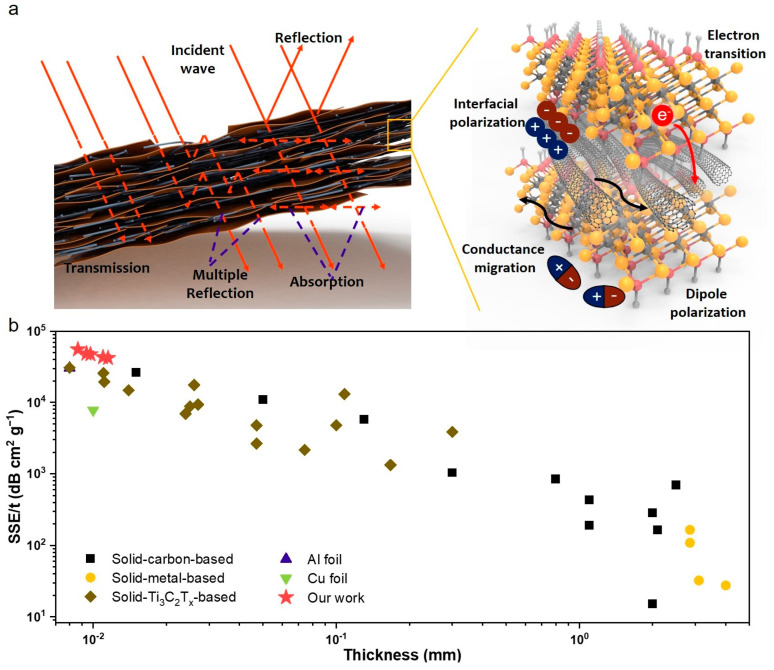
(**a**) EMI shielding schematic of MWCNT/Ti_3_C_2_T_x_ hybrid films. (**b**) Comparison of the SSE/t as a function of the thickness of different EMI shielding materials.

A potential EMI shielding mechanism of the MWCNT/Ti_3_C_2_T_x_ hybrid films is proposed as illustrated in Figure 6a, where a large number of electromagnetic waves directly reflect the free space due to the impedance mismatch between free space and hybrid films, and only a small amount of electromagnetic waves enter the interior of the hybrid films for absorption and multiple reflections, which can be attributed to the synergistic interactions of the MWCNT and Ti_3_C_2_T_x_ with high conduction loss and dipole polarization loss to the electromagnetic waves and multiple reflections of incident electromagnetic waves within the lamellar structure.

Considering the low thickness and density of the MWCNT/Ti_3_C_2_T_x_ hybrid films, the SSE/t values were compared with other typical shielding materials, as shown in Figure 6b. Compared with typical carbon-based [31,32,33,34,35,36,37,38,39,40], metal-based [7,22], and Ti_3_C_2_T_x_-based [22,25,41,42,43,44,45] materials, the obtained MWCNT/Ti_3_C_2_T_x_ hybrid films presented a high SSE/t of 55,603.1 dB∙cm^2^∙g^−1^ with a thickness of 8.6 μm. More detailed information in Table S1 shows the comparisons of EMI shielding performance for these EMI shielding materials. It can be found that MWCNT/Ti_3_C_2_T_x_ hybrid films have obvious advantages of low thickness and high SSE/t, which demonstrates the potential application of EMI shielding in flexible electronic and other function materials.

## 4. Conclusions

The hybridization of Ti_3_C_2_T_x_ nanosheets and carbon nanotubes promoted the formation of an ordered lamellar assembly structure and a complete conductive path, which resulted in obvious improvement both in conductivity (452.5 S/cm) and EMI SE (44.3 dB) for MWCNT/Ti_3_C_2_T_x_ hybrid film (50 wt% Ti_3_C_2_T_x_). In addition, the hybrid film was endowed with low thickness (8.6 μm) and high SSE/t (55,603.1 dB∙cm^2^∙g^−1^). Compared with pure carbon nanotube films, the high conductivity and lamellar structure brought about excellent improvement in EMI SE at 41.5%, which possesses broad application prospects in flexible devices and aerospace fields.

## Data Availability

The original contributions presented in this study are included in the article and Supplementary Material. Further inquiries can be directed to the corresponding author.

## Supplementary Materials

The following supporting information can be downloaded at https://www.mdpi.com/article/10.3390/nano15010006/s1, Table S1: Comparison of the SSE/t as a function of the thickness between MWCNT/Ti_3_C_2_T_x_ hybrid films and other solid EMI shielding materials. References [7,22,25,31,32,33,34,35,36,37,38,39,40,41,42,43,44,45] are cited in the supplementary materials.
