# Supplementary material for: Ultrathin MWCNT/Ti3C2Tx Hybrid Films for Electromagnetic Interference Shielding
Source: Nanomaterials (Basel). 2024 Dec 25;15(1):6. doi: 10.3390/nano15010006 (PMC11721604; doi:10.3390/nano15010006)
Supplement: Supplementary file 1 [file nanomaterials-15-00006-s001.zip › nanomaterials-3359102-supplementary.pdf]

Table S1. Comparison of the SSE/*t* as a function of the thickness between MWCNT/$Ti_3C_2T_x$ hybrid films and other solid EMI shielding materials [1-18].

(rGO: reduced graphene oxide, CNT: carbon nanotube, CB: carbon black, PS: polystyrene, PEDOT:PSS: poly(3,4-ethylenedioxythiophene)–poly(styrenesulfonate), PC: polycarbonate, ABS: acrylonitrile-butadiene-styrene, EPDM: ethylene-propylene diene monomer, SA: sodium alginate, PVA: polyvinyl alcohol, CNF: cellulose nanofiber, TOCNF: 2,2,6,6-tetramethyl-1-piperidinyloxy oxidized cellulose nanofiber, PI: polyimide.)

Types	Materails	Thickness (mm)	EMI (dB)	SSE/*t* (dB·cm^2·g^{-1})	References
**Carbon-based**	rGO/PS	2.5	45.1	692	1
	rGO/Fe_3O_4	0.3	24	1033	2
	Graphene/PEDOT:PSS	0.8	70	841	3
	MWCNT/PC	2.1	39	164	4
	MWCNT/ABS	1.1	50	432.7	5
	CB/ABS	1.1	20	190	
	MWCNT/PS	2	30	285	6
	CB/EPDM	2	18	15.1	7
	Graphene/CNT film	0.015	57.6	26483	8
	MWCNT buckpaper	0.13	43	5803	9
	MWCNT nanopaper	0.05	30	10909	10
**Metal-based**	Copper	3.1	90	32.3	11
	Stainless steel	4	89	27.5	
	Ni Fiber	2.85	58	108.7	
	Ni filaments	2.85	87	164.9	
	Al foil	0.008	66	30555	12
	Cu foil	0.01	70	7812	
**$Ti_3C_2T_x$-based**	$Ti_3C_2T_x$	0.011	68	25863	12
	$Ti_3C_2T_x$/SA	0.008	57	30830	
	$Ti_3C_2T_x$/PEDOT:PSS	0.0111	42.1	19497.8	13
	PVA/MXene	0.024	26.9	6919	14
		0.025	37.1	8833	
		0.027	44.4	9343	
	$Ti_3C_2T_x$/SA	0.014	43.9	14830	15
		0.026	54.3	17586	
	$Ti_3C_2T_x$/CNF	0.167	25	1326	16
		0.074	26	2154	

		0.047	24	2647	
	$Ti_3C_2T_x$/TOCNF	0.047	32.7	4761	17
	PI-$Ti_3C_2T_x$-MWCNT	0.108	66.8	13153.8	18
	MWCNT/$Ti_3C_2T_x$	0.0115	33.5	42029.9	This work
		0.011	34.4	43218.9	
		0.0097	37.8	47510.3	
		0.0094	38.8	48695.1	
		0.0086	44.3	55603.1	

References:

1. Yan, D.-X.; Pang, H.; Li, B.; Vajtai, R.; Xu, L.; Ren, P.-G.; Wang, J.-H.; Li, Z.-M. Structured Reduced Graphene Oxide/Polymer Composites for Ultra-Efficient Electromagnetic Interference Shielding. *Adv. Funct. Mater.* **2015**, *25*, 559–566. https://doi.org/10.1002/adfm.201403809.
2. Song, W.-L.; Guan, X.-T.; Fan, L.-Z.; Cao, W.-Q.; Wang, C.-Y.; Zhao, Q.-L.; Cao, M.-S. Magnetic and conductive graphene papers toward thin layers of effective electromagnetic shielding. *J. Mater. Chem. A* **2015**, *3*, 2097–2107. https://doi.org/10.1039/c4ta05939e.
3. Agnihotri, N.; Chakrabarti, K.; De, A. Highly efficient electromagnetic interference shielding using graphite nanoplatelet/poly(3,4-ethylenedioxythiophene)–poly(styrenesulfonate) composites with enhanced thermal conductivity. *RSC Adv.* **2015**, *5*, 43765–43771. https://doi.org/10.1039/c4ra15674a.
4. Pande, S.; Chaudhary, A.; Patel, D.; Singh, B.P.; Mathur, R.B. Mechanical and electrical properties of multiwall carbon nanotube/polycarbonate composites for electrostatic discharge and electromagnetic interference shielding applications. *RSC Adv.* **2014**, *4*, 13839–13849. https://doi.org/10.1039/c3ra47387b.
5. Al-Saleh, M.H.; Saadeh, W.H.; Sundararaj, U. EMI shielding effectiveness of carbon based nanostructured polymeric materials: A comparative study. *Carbon* **2013**, *60*, 146–156. https://doi.org/10.1016/j.carbon.2013.04.008.
6. Arjmand, M.; Apperley, T.; Okoniewski, M.; Sundararaj, U. Comparative study of electromagnetic interference shielding properties of injection molded versus compression molded multi-walled carbon nanotube/polystyrene composites. *Carbon* **2012**, *50*, 5126–5134. https://doi.org/10.1016/j.carbon.2012.06.053.
7. Ghosh, P.; Chakrabarti, A. Conducting carbon black filled EPDM vulcanizates: Assessment of dependence of physical and mechanical properties and conducting character on variation of filler loading. *Eur. Polym. J.* **2000**, *36*, 1043–1054. https://doi.org/10.1016/S0014-3057(99)00157-3.
8. Zhou, E.; Xi, J.; Guo, Y.; Liu, Y.; Xu, Z.; Peng, L.; Gao, W.; Ying, J.; Chen, Z.; Gao, C. Synergistic effect of graphene and carbon nanotube for high-performance electromagnetic interference shielding films. *Carbon* **2018**, *133*, 316–322. https://doi.org/10.1016/j.carbon.2018.03.023.
9. Lu, S.; Shao, J.; Ma, K.; Chen, D.; Wang, X.; Zhang, L.; Meng, Q.; Ma, J. Flexible, mechanically resilient carbon nanotube composite films for high-efficiency electromagnetic interference shielding. *Carbon* **2018**, *136*, 387–394. https://doi.org/10.1016/j.carbon.2018.04.086.
10. Zhang, D.; Villarreal, M.G.; Cabrera, E.; Benatar, A.; James Lee, L.; Castro, J.M. Performance study of ultrasonic assisted processing of CNT nanopaper/solventless epoxy composite. *Compos. Part B Eng.* **2019**, *159*, 327–335. https://doi.org/10.1016/j.compositesb.2018.10.012.
11. Shui, X.; Chung, D.D.L. Nickel filament polymer-matrix composites with low surface impedance and high electromagnetic interference shielding effectiveness. *J. Electron. Mater.* **1997**, *26*, 928–934. https://doi.org/10.1007/s11664-997-0276-4.
12. Shahzad, F.; Alhabeb, M.; Hatter, C.B.; Anasori, B.; Man Hong, S.; Koo, C.M.; Gogotsi, Y. Electromagnetic interference shielding with 2D transition metal carbides (MXenes). *Science* **2016**, *353*, 1137–1140. https://doi.org/10.1126/science.aag2421.
13. Liu, R.; Miao, M.; Li, Y.; Zhang, J.; Cao, S.; Feng, X. Ultrathin Biomimetic Polymeric $Ti_3C_2T_x$ MXene Composite Films for Electromagnetic Interference Shielding. *ACS Appl. Mater. Interfaces* **2018**, *10*, 44787–44795. https://doi.org/10.1021/acsami.8b18347.
14. Jin, X.X.; Wang, J.F.; Dai, L.Z.; Liu, X.Y.; Li, L.; Yang, Y.Y.; Cao, Y.X.; Wang, W.J.; Wu, H.; Guo, S.Y. Flame-retardant poly(vinyl alcohol)/MXene multilayered films with outstanding electromagnetic interference shielding and thermal conductive performances. *Chem. Eng. J.* **2020**, *380*, 122475. https://doi.org/10.1016/j.cej.2019.122475.

15. Zhou, Z.; Liu, J.; Zhang, X.; Tian, D.; Zhan, Z.; Lu, C. Ultrathin MXene/Calcium Alginate Aerogel Film for High-Performance Electromagnetic Interference Shielding. *Adv. Mater. Interfaces* **2019**, *6*, 1802040. https://doi.org/10.1002/admi.201802040.
16. Cao, W.T.; Chen, F.F.; Zhu, Y.J.; Zhang, Y.G.; Jiang, Y.Y.; Ma, M.G.; Chen, F. Binary Strengthening and Toughening of MXene/Cellulose Nanofiber Composite Paper with Nacre-Inspired Structure and Superior Electromagnetic Interference Shielding Properties. *ACS Nano* **2018**, *12*, 4583–4593. https://doi.org/10.1021/acsnano.8b00997.
17. Zhan, Z.Y.; Song, Q.C.; Zhou, Z.H.; Lu, C.H. Ultrastrong and conductive MXene/cellulose nanofiber films enhanced by hierarchical nano-architecture and interfacial interaction for flexible electromagnetic interference shielding. *J. Mater. Chem. C* **2019**, *7*, 9820–9829. https://doi.org/10.1039/c9tc03309b.
18. Liang, W.; Wu, J.; Zhang, S.; Zhao, P.-Y.; Zuo, X.; Wang, G.-S. Construction of PI-MXene-MWCNT nanocomposite film integrating conductive gradient with sandwich structure for high-efficiency electromagnetic interference shielding in extreme environments. *Carbon* **2024**, *228*, 119328. https://doi.org/10.1016/j.carbon.2024.119328.
